# Serum 25-hydroxyvitamin D and postmenopausal breast cancer survival: a prospective patient cohort study

**DOI:** 10.1186/bcr2920

**Published:** 2011-07-26

**Authors:** Alina Vrieling, Rebecca Hein, Sascha Abbas, Andreas Schneeweiss, Dieter Flesch-Janys, Jenny Chang-Claude

**Affiliations:** 1 Division of Cancer Epidemiology, German Cancer Research Center, Im Neuenheimer Feld 581, 69120 Heidelberg, Germany; 2 PMV Research Group at the Department of Child and Adolescent Psychiatry and Psychotherapy, University of Cologne, Herderstrasse 52-54, 50931 Cologne, Germany; 3 Section Gynecological Oncology, National Center for Tumor Diseases, University Hospital Heidelberg, Im Neuenheimer Feld 460, 69120 Heidelberg, Germany; 4 Department of Cancer Epidemiology/Clinical Cancer Registry, University Cancer Center Hamburg (UCCH) and Department of Medical Biometrics and Epidemiology, University Medical Center Hamburg-Eppendorf, Martinistrasse 52, 20246 Hamburg, Germany

## Abstract

**Introduction:**

Vitamin D has been postulated to be involved in cancer prognosis. Thus far, only two studies reported on its association with recurrence and survival after breast cancer diagnosis yielding inconsistent results. Therefore, the aim of our study was to assess the effect of post-diagnostic serum 25-hydroxyvitamin D [25(OH)D] concentrations on overall survival and distant disease-free survival.

**Methods:**

We conducted a prospective cohort study in Germany including 1,295 incident postmenopausal breast cancer patients aged 50-74 years. Patients were diagnosed between 2002 and 2005 and median follow-up was 5.8 years. Cox proportional hazards models were stratified by age at diagnosis and season of blood collection and adjusted for other prognostic factors. Fractional polynomials were used to assess the true dose-response relation for 25(OH)D.

**Results:**

Lower concentrations of 25(OH)D were linearly associated with higher risk of death (hazard ratio (HR) = 1.08 per 10 nmol/L decrement; 95% confidence interval (CI), 1.00 to 1.17) and significantly higher risk of distant recurrence (HR = 1.14 per 10 nmol/L decrement; 95%CI, 1.05 to 1.24). Compared with the highest tertile (≥ 55 nmol/L), patients within the lowest tertile (< 35 nmol/L) of 25(OH)D had a HR for overall survival of 1.55 (95%CI, 1.00 to 2.39) and a HR for distant disease-free survival of 2.09 (95%CI, 1.29 to 3.41). In addition, the association with overall survival was found to be statistically significant only for 25(OH)D levels of blood samples collected before start of chemotherapy but not for those of samples taken after start of chemotherapy (*P *for interaction = 0.06).

**Conclusions:**

In conclusion, lower serum 25(OH)D concentrations may be associated with poorer overall survival and distant disease-free survival in postmenopausal breast cancer patients.

## Introduction

Vitamin D can be obtained from exposure to sunlight and through diet and supplements. Low vitamin D levels have been inconsistently associated with increased breast cancer risk [1]. The anticarcinogenic potential of vitamin D is attributed to the active or hormonal form of vitamin D, 1,25-dihydroxyvitamin D (1,25(OH)_2_D), which is produced in the kidneys from the metabolite, 25-hydroxyvitamin D (25(OH)D) [2]. As 1,25(OH)_2_D is homeostatically tightly regulated, 25(OH)D is a better indicator of vitamin D status from both sunlight exposure and ingested vitamin D over longer periods [3].

It is currently unclear whether vitamin D affects breast cancer survival, although some circumstantial evidence exists. Several ecological studies in both the USA and Europe have reported that breast cancer mortality rates are inversely associated with total solar or ultraviolet B irradiance [4-6]. Two studies in Norway and England found that breast cancer mortality was lowest for cancers diagnosed in summer and autumn, the seasons with the highest concentrations of 25(OH)D [7-9]. Lower serum 25(OH)D levels have also been associated with more advanced stages of breast cancer. An observational study among Caucasian women showed that serum levels of 25(OH)D were lower in patients with locally advanced or metastatic breast cancer than in those with early-stage disease [10]. In a multi-ethnic cohort of breast cancer survivors, women with localized or regional breast cancer had lower serum 25(OH)D levels than those with *in situ *disease [11].

To our knowledge, only two studies examined the association between measured post-diagnostic circulating 25(OH)D concentrations and breast cancer recurrence and survival [12,13]. A prospective cohort study in 512 breast cancer patients reported that vitamin D deficiency may be associated with increased risk of distant recurrence and death [12]. However, this finding was not confirmed in a recent nested case-control study comparing 512 matched pairs of breast cancer patients with and without recurrence [13]. Thus, we examined post-diagnostic serum concentrations of 25(OH)D in relation to overall and distant disease-free survival in a large prospective cohort of 1,295 postmenopausal breast cancer patients.

## Materials and methods

### Study population

Patients were recruited from 2002 to 2005 within a large population-based case-control study on breast cancer in two regions in Germany (MARIE study, Mamma Carcinoma Risk factor Investigation) [14], and a follow up was performed up to the end of 2009. Individuals in this analysis were patients with histologically confirmed primary invasive (stage I to IV) or *in situ *breast cancer diagnosed between 1 August 2002 and 31 July 2005 in the Rhein-Neckar-Karlsruhe region. Patients were identified through frequent monitoring of hospital admissions, surgery schedules and pathology records. Patients were aged between 50 and 74 years and postmenopausal (defined as last menstrual bleeding at least 12 months before the date of diagnosis, a bilateral oophorectomy, cessation of menses due to radiation or chemotherapy, > 55 years with unclear menopausal status due to hysterectomy or hormone use). Post-diagnostic serum samples were available for 1,385 patients. After exclusion of patients with previous cancer (other than basal or squamous skin cancers or *in situ *cancers) or missing information on previous cancer (*n *= 90), our final study population consisted of 1,295 postmenopausal breast cancer patients.

This study was approved by the ethics committees of both the University of Heidelberg and the University of Hamburg and conducted in accordance with the Declaration of Helsinki. All study participants provided informed consent.

### Exposure assessment

Serum was collected after cancer diagnosis and stored in aliquots at -80°C until measurement. Samples were previously analyzed in a single batch between November 2006 and January 2007 [15]. For quantification of 25(OH)D in serum, the OCTEIA 25(OH)D enzyme immunoassay (Immunodiagnostic Systems Limited, Boldon, UK) was used. The intra-assay and inter-assay coefficient of variation were 3.4% and 7.6%, respectively.

Clinical and pathological characteristics were abstracted from hospital and pathology records. All patients were interviewed at recruitment (2002 to 2005) by trained personnel to obtain information on sociodemographic factors, anthropometric measures, lifetime hormone replacement therapy (HRT) exposure, and other potential breast cancer risk factors.

### Outcome assessment

Vital status of participants was determined through population registries up to the end of 2009, and all deaths were verified by death certificates. Recurrences of the primary breast cancer or second cancers were identified during a telephone interview conducted from May to September 2009. Further, for deceased participants and those not participating in the telephone interview, medical records were checked or treating physicians were contacted. All recurrences or second cancers were verified by clinical records or through records from treating physicians. Primary outcomes were overall survival and distant disease-free survival (including distant recurrence, death, second primary invasive non-breast cancer), secondary outcomes were breast cancer-specific survival and recurrence-free survival (including ipsilateral/local/regional invasive recurrence, distant recurrence, death) [16]. Analyses for distant disease-free survival and recurrence-free survival were restricted to participants with *in situ *breast cancer or stage I to IIIa disease (*n *= 1,088). Participants were censored at date of last contact or 31 December 2009, whichever came first.

### Statistical analyses

Cox proportional hazards models were used to examine the association of serum 25(OH)D with overall survival and distant disease-free survival as primary endpoints, and breast cancer-specific survival and recurrence-free survival as secondary endpoints. Hazard ratios (HR) and 95% confidence intervals (CIs) were calculated using 25(OH)D concentration both as continuous (per 10 nmol/L decrement) and as categorical variable divided into three categories (< 35 nmol/L, 35 to 55 nmol/L, ≥ 55 nmol/L), closely corresponding to the classification into tertiles. The highest exposure category was defined as the reference category. Follow-up time was used as the time variable, and calculated as the time between the date of diagnosis and the date of event or censoring. All analyses were stratified by age at diagnosis (in one-year categories) and season of serum collection (January to March, April to June, July to September, October to December). Analyses were adjusted for the traditional prognostic variables, that is, tumor size (*in situ*, neoadjuvant chemotherapy, ≤ 2 cm, 2 to 5 cm, > 5 cm, growth in chest wall/skin), nodal status (*in situ*, neoadjuvant chemotherapy, 0, 1 to 3, 4 to 9, ≥ 10), metastasis (*in situ*, yes, no), tumor grade (*in situ*, neoadjuvant chemotherapy, low + moderate, high), and joint estrogen receptor (ER)/progesterone receptor (PR) status (*in situ*, neoadjuvant chemotherapy, ER^+^PR^+^, ER^+^PR^-^/ER^-^PR^+^, ER^-^PR^-^). In addition, analyses were adjusted for variables that were statistically significant (< 0.05) when tested in the model, that is, self-reported prevalent diabetes (yes, no) and mode of detection (physician-detected by routine investigation/mammography/ultrasound, self-detected by palpation/secretion/pain). Other potentially confounding variables were not statistically significant and did not change the risk estimates by 10% or more when tested in the model and were therefore not included in the final model, that is, human epidermal growth factor receptor 2 (HER2) status, type of surgery, chemotherapy, radiotherapy, hormonal therapy, HRT use, body mass index (BMI) at time of recruitment, leisure time physical activity since age 50 years, cardiovascular disease, and time between diagnosis and blood collection. Tests for linear trend with log HR were performed using 25(OH)D concentration as a continuous variable. We used the method of fractional polynomials to further examine dose-response relation and non-linearity of the log HR for 25(OH)D concentrations [17]. The continuous 25(OH)D variable was entered into the multivariate cox proportional hazards model via a set of defined transformations (*x*^-2^, *x*^-1^, *x*^-0.5^, *x*^0.5^, *x*^2^, *x*^3 ^and log(*x*)), allowing a maximum of two terms (including the untransformed variable) in the model. The function that best fitted the data was selected on the basis of the -2 log likelihood of the respective model.

We performed sensitivity analyses by time between diagnosis and blood collection and by time of blood collection relative to start of chemotherapy (blood collection before start of chemotherapy or non-chemotherapy *vs *blood collection after start of chemotherapy).

We performed stratified analyses to examine whether the associations between 25(OH)D and survival varied by BMI at time of recruitment (less than *vs *median or above kg/m^2^), leisure time physical activity since age 50 (less than *vs *median or above median metabolic equivalent (MET)-h/wk), HRT use (never/past *vs *current), and ER status (ER^+ ^*vs *ER^-^). We then included interaction terms of the continuous 25(OH)D concentration variable and the variables of interest in the fully adjusted model and evaluated statistical significance with the likelihood ratio test.

All tests were two-sided and considered to be statistically significant if *P *value was less than 0.05. All statistical analyses were performed using SAS software 9.2 (SAS Institute, Cary, NC, USA).

## Results

Mean (standard deviation) age at diagnosis was 63.4 ± 5.5 years. Serum samples were drawn a median of 83 days after diagnosis (range 2 to 1,112 days), and the median 25(OH)D level was 44.9 nmol/L (range 9.7 to 240.3 nmol/L).

Baseline characteristics according to tertiles of serum 25(OH)D are shown in Table 1. Participants with lower 25(OH)D levels were diagnosed at a somewhat older age, had a shorter time interval between diagnosis and blood collection, a higher BMI at time of recruitment, and less leisure time physical activity since age 50 years compared with participants with higher 25(OH)D levels. Participants in the lowest tertile of serum 25(OH)D were more likely to have their blood collected in winter. Further, they had a higher proportion of tumors with a larger tumor size, more lymph node involvement, metastases, and a higher tumor grade, and a lower proportion of ER^-^PR^- ^tumors. They were also more likely to be treated with chemotherapy, not to use HRT, to have diabetes or cardiovascular disease, and to have a self-detected tumor. No clear differences in radiotherapy or tamoxifen/aromatase inhibitor use by tertiles of serum 25(OH)D were observed.

**Table 1:** Baseline characteristics of study cohort according to tertiles of serum 25(OH)D (*n *= 1,295)

	Serum 25(OH)D		
Characteristic	< 35 nmol/L	35-55 nmol/L	≥ 55 nmol/L
Number of patients	432	407	456
Age at diagnosis (years), mean ± SD	64.0 ± 5.7	63.7 ± 5.4	62.5 ± 5.4
25(OH)D concentration (nmol/L), median	25.7	44.4	71.3
Follow-up time (years), median	5.6	5.9	6.0
Time between diagnosis and blood collection (days), median	82	63	130
BMI at recruitment (kg/m^2^), mean ± SD	27.7 ± 4.9	26.6 ± 4.4	25.5 ± 4.1
Leisure time physical activity since age 50 (met-hr/wk), mean ± SD	37.3 ± 30.0	43.5 ± 33.0	49.5 ± 36.4
Season of blood draw, %			
Jan-Mar	37.5	16.7	13.6
Apr-Jun	22.0	24.8	16.0
Jul-Sept	13.0	29.5	43.6
Oct-Dec	27.5	29.0	26.8
Tumor size, %			
*In situ*	3.9	4.9	8.3
≤ 2 cm	44.4	51.8	51.1
> 2- ≤ 5 cm	32.9	32.4	32.2
> 5 cm	5.6	2.2	2.6
Growth into chest wall/skin	5.6	2.0	1.6
Neoadjuvant CT	7.4	6.1	4.2
Missing	0.2	0.5	0.0
Nodal status, %*			
0	54.4	58.2	59.6
1-3	19.2	19.7	19.7
4-9	6.7	6.6	4.0
≥ 10	7.6	3.7	4.2
Missing	0.7	0.7	0.0
Metastases, %			
*In situ*	3.9	4.9	8.3
No	89.1	89.7	88.8
Yes	5.6	4.9	2.2
Missing	1.4	0.5	0.7
Tumor grade, %*			
Low + moderate	61.1	65.1	64.5
High	27.3	23.3	22.8
Missing	0.2	0.5	0.2
ERPR, %*			
ER^+^PR^+^	55.3	55.0	50.9
ER^+^PR^-^/ER^-^PR^+^	19.2	15.7	20.8
ER^-^PR^-^	14.1	17.4	15.8
Missing	0.0	0.7	0.0
Chemotherapy, %			
No	49.1	52.1	58.8
Yes	48.8	47.6	41.0
Missing	2.1	0.3	0.2
Radiotherapy, %			
No	27.8	17.9	25.4
Yes	72.0	82.1	74.4
Missing	0.2	0.0	0.2
Tamoxifen/aromatase inhibitor use, %			
No	14.8	18.9	19.3
Yes	79.9	77.2	76.3
Missing	5.3	3.9	4.4
Hormone replacement therapy, %			
No	71.1	57.7	48.9
Yes	27.8	41.5	50.0
Missing	1.1	0.7	1.1
Diabetes, %			
No	87.0	89.7	91.9
Yes	13.0	10.3	7.9
Missing	0.0	0.0	0.2
Cardiovascular disease, %			
No	41.0	48.2	51.8
Yes	59.0	51.8	48.2
Mode of detection, %			
Self-detected by palpation/secretion/pain	65.3	56.3	53.5
Physician-detected by routine investigation/mammography/ultrasound	34.5	43.5	45.4
Missing	0.2	0.2	1.1

25(OH)D, 25-hydroxyvitamin D; BMI, body mass index; CT, chemotherapy; ERPR, estrogen receptor/progesterone receptor; SD, standard deviation.

* Includes a separate category for *in situ *and neoadjuvant CT.

Overall, median follow-up time was 5.8 years (range 0.2 to 7.7 years) and 183 deaths occurred, 137 due to breast cancer. Other causes of death were other cancers (*n *= 16), cardiovascular disease (*n *= 18), and other causes (*n *= 12). Of the patients with *in situ *breast cancer or stage I to IIIa disease, 137 women experienced an event related to distant disease-free survival and 145 women experienced an event related to recurrence-free survival.

We assessed the association of post-diagnostic serum 25(OH)D with survival. Results for overall survival and breast cancer-specific survival and results for distant disease-free survival and recurrence-free survival were similar: overall survival and distant disease-free survival results are presented here (Table 2). A trend towards increased risk of death was seen, with an adjusted HR of 1.08 (95% CI, 1.00 to 1.17; *P *for trend = 0.07) per 10 nmol/L decrement in 25(OH)D. The adjusted HR for the highest compared with the lowest tertile of 25(OH)D was 1.55 (95% CI, 1.00 to 2.39). Restriction to participants with stage I to IIIa disease provided similar results (per 10 nmol/L decrement: HR = 1.11; 95% CI, 0.99 to 1.23; *P *for trend = 0.08, lowest *vs *highest tertile: HR = 1.67; 95% CI, 0.91 to 3.06; data not shown). The adjusted HR for risk of distant disease was significantly increased (per 10 nmol/L increment: HR = 1.14; 95% CI, 1.05 to 1.24; *P *for trend = 0.006, lowest *vs *highest tertile: HR = 2.09; 95% CI, 1.29 to 3.41). We further examined the shape of the association using fractional polynomials and found a linear association between the log HR and the 25(OH)D concentration for both overall survival and distant disease-free survival (data not shown).

**Table 2:** Hazard ratios of overall mortality and distant disease according to tertiles of serum 25(OH)D

	Serum 25(OH)D												
	Continuous				Categorized								
	per 10 nmol/L decrement				< 35 nmol/L			35-55 nmol/L			≥ 55 nmol/L		
Outcome	No. (events)	HR	95% CI	*P*_trend_^a^	No. (events)	HR	95% CI	No. (events)	HR	95% CI	No. (events)	HR	95% CI

Overall mortality													
Crude†^b^	1,295 (183)	1.11	1.03 to 1.18	0.009	432 (87)	1.96	1.32 to 2.90	407 (46)	0.94	0.62 to 1.44	456 (50)	1.00	-
Multivariate‡^c^	1,265 (174)	1.08	1.00 to 1.17	0.07	422 (82)	1.55	1.00 to 2.39	397 (44)	0.72	0.45 to 1.17	446 (48)	1.00	-
Distant disease													
Crude^b^	1,088 (137)	1.12	1.03 to 1.21	0.01	341 (64)	2.09	1.31 to 3.30	347 (37)	1.19	0.73 to 1.93	400 (36)	1.00	-
Multivariate^c^	1,074 (135)	1.14	1.05 to 1.24	0.006	338 (63)	2.09	1.29 to 3.41	342 (37)	1.16	0.70 to 1.94	394 (35)	1.00	-

25(OH)D, 25-hydroxyvitamin D; CI, confidence interval; HR, hazard ratio.

^a ^Calculated by using serum 25(OH)D as a continuous variable.

^b ^Stratified by age at diagnosis and season.

^c ^Stratified by age at diagnosis and season, adjusted for tumor size, nodal status, metastases, tumor grade, estrogen/progesterone receptor status, diabetes, mode of detection; due to missing covariate values, 30 observations were not included in the multivariate model.

To evaluate whether the association between serum 25(OH)D and survival depends on the time interval between cancer diagnosis and blood collection, we restricted our analyses to a time interval of 2, 1, and 0.5 years (Table 3). The HRs for death were somewhat larger with a shorter period between cancer diagnosis and blood collection. However, there was no significant difference for risk of death and for distant disease by time of blood collection after cancer diagnosis.

**Table 3:** Hazard ratios^a ^according to tertiles of serum 25(OH)D and time between diagnosis and blood collection

	Serum 25(OH)D												
	Continuous				Categorized								
	per 10 nmol/L decrement				< 35 nmol/L			35-55 nmol/L			≥ 55 nmol/L		
Outcome	**No**. (events)	HR	95% CI	*P*_trend_^b^	**No**. (events)	HR	95% CI	**No**. (events)	HR	95% CI	**No**. (events)	HR	95% CI
Overall mortality													
All	1,265 (174)	1.08	1.00 to 1.17	0.07	422 (82)	1.55	1.00 to 2.39	397 (44)	0.72	0.45 to 1.17	446 (48)	1.00	-
< 2 years	1,249 (172)	1.09	1.00 to 1.17	0.05	418 (82)	1.57	1.01 to 2.43	394 (44)	0.75	0.46 to 1.22	437 (46)	1.00	-
< 1 year	1,093 (151)	1.13	1.03 to 1.22	0.02	375 (77)	1.81	1.11 to 2.94	353 (39)	0.82	0.48 to 1.42	365 (35)	1.00	-
< 0.5 year	807 (119)	1.15	1.03 to 1.26	0.02	277 (63)	2.19	1.21 to 3.97	275 (33)	0.96	0.50 to 1.83	255 (23)	1.00	-
Distant disease													
All	1,074 (135)	1.14	1.05 to 1.24	0.006	338 (63)	2.09	1.29 to 3.41	342 (37)	1.16	0.70 to 1.94	394 (35)	1.00	-
< 2 years	1,059 (133)	1.15	1.05 to 1.25	0.005	335 (63)	2.11	1.30 to 3.45	339 (37)	1.17	0.70 to 1.95	385 (33)	1.00	-
< 1 year	925 (119)	1.15	1.05 to 1.26	0.007	298 (59)	2.24	1.32 to 3.79	302 (32)	1.28	0.74 to 2.22	325 (28)	1.00	-
< 0.5 year	685 (95)	1.13	1.01 to 1.25	0.05	219 (48)	2.16	1.18 to 3.98	235 (25)	1.12	0.60 to 2.10	231 (22)	1.00	-

25(OH)D, 25-hydroxyvitamin D; CI, confidence interval; HR, hazard ratio.

^a ^Stratified by age at diagnosis and season, adjusted for tumor size, nodal status, metastases, tumor grade, estrogen/progesterone receptor status, diabetes, mode of detection.

^b ^Calculated by using serum 25(OH)D as a continuous variable.

Given that serum levels of 25(OH)D could be affected by chemotherapy, we investigated whether the association with survival differed for participants that did not receive chemotherapy before blood collection and participants with blood collection after start of chemotherapy (Table 4). The association with risk of death remained significant only for participants that did not receive chemotherapy before blood collection (per 10 nmol/L decrement: HR = 1.15; 95% CI, 1.03 to 1.27; *P *for trend = 0.02) whereas no association was found for participants with blood collection after start of chemotherapy (*P *for interaction = 0.06). However, for risk of distant disease no such heterogeneity was observed (*P *for interaction = 0.49).

**Table 4:** Hazard ratios^a ^according to tertiles of serum 25(OH)D and chemotherapy

	Serum 25(OH)D												
	Continuous				Categorized								
	per 10 nmol/L decrement				< 35 nmol/L			35-55 nmol/L			≥ 55 nmol/L		
Outcome	**No**. (events)	HR	95% CI	*P*_trend_^b^	**No**. (events)	HR	95% CI	**No**. (events)	HR	95% CI	**No**. (events)	HR	95% CI
Overall mortality													
All	1,265 (174)	1.08	1.00 to 1.17	0.07	422 (82)	1.55	1.00 to 2.39	397 (44)	0.72	0.45 to 1.17	446 (48)	1.00	-
No/before CT^c^	911 (99)	1.15	1.03 to 1.27	0.02	292 (51)	2.17	1.18 to 3.99	276 (20)	1.00	0.51 to 1.96	343 (28)	1.00	-
After CT^d^	339 (71)	0.91	0.75 to 1.08	0.27	121 (29)	0.82	0.35 to 1.95	117 (22)	0.59	0.24 to 1.44	101 (20)	1.00	-
Distant disease													
All	1,074 (135)	1.14	1.05 to 1.24	0.006	338 (63)	2.09	1.29 to 3.41	342 (37)	1.16	0.70 to 1.94	394 (35)	1.00	-
No/before CT^c^	844 (103)	1.17	1.06 to 1.29	0.005	259 (50)	2.41	1.37 to 4.22	263 (26)	1.07	0.59 to 1.94	322 (27)	1.00	-
After CT^d^	221 (32)	1.12	0.85 to 1.39	0.41	73 (13)	1.79	0.35 to 9.00	78 (11)	0.84	0.14 to 4.97	70 (8)	1.00	-

Note: 18 and 9 participants, respectively, had missing data on chemotherapy and were excluded from the stratified analyses.

25(OH)D, 25-hydroxyvitamin D; CI, confidence interval; CT, chemotherapy; HR, hazard ratio.

^a ^Stratified by age at diagnosis and season, adjusted for tumor size, nodal status, metastases, tumor grade, estrogen/progesterone receptor status, diabetes, mode of detection

^b ^Calculated by using serum 25(OH)D as a continuous variable.

^c ^No chemotherapy or blood collection before start chemotherapy.

^d ^Blood collection after start chemotherapy.

*P*_interaction _= 0.06 and 0.49 for overall mortality and distant disease, respectively.

We further examined the association for serum 25(OH)D across strata of other potential predictors of cancer recurrence and mortality (Table 5). Associations tended to be stronger among participants that had a higher BMI at time of recruitment, higher leisure time physical activity since age 50 years, and were never or past users of HRT. However, statistical power was diminished, and none of the *P*-values for interaction were statistically significant.

**Table 5:** Multivariate-adjusted^a ^stratified analyses of overall mortality according to tertiles of serum 25(OH)D

		Serum 25(OH)D								
		Continuous			Categorical					
		per 10 nmol/L decrement			< 35 nmol/L		35-55 nmol/L		≥ 55 nmol/L	
Variable	**Total no**.	HR	95%CI	*P*_trend_^b^	HR	95%CI	HR	95%CI		*P*_interaction_^c^
BMI, kg/m^2d^										0.74
< 26	382	1.05	0.66 to 1.44	0.81	0.64	0.08 to 5.00	0.02	0.00 to 2.06	Referent	
≥ 26	393	1.15	0.94 to 1.36	0.20	2.57	0.88 to 7.54	0.60	0.19 to 1.92	Referent	
Leisure time physical activity, MET-h/wk										0.49
< 37	631	1.04	0.92 to 1.17	0.50	1.19	0.62 to 2.29	0.63	0.30 to 1.32	Referent	
≥ 37	629	1.19	1.03 to 1.35	0.03	1.90	0.85 to 4.24	0.57	0.22 to 1.48	Referent	
HRT use										0.40
Never, past	746	1.13	1.01 to 1.25	0.04	1.89	1.09 to 3.26	0.61	0.31 to 1.19	Referent	
Current	506	1.03	0.86 to 1.21	0.73	0.89	0.26 to 3.05	1.17	0.46 to 2.98	Referent	
ER status										0.78
ER^+^	903	1.08	0.97 to 1.19	0.17	1.70	0.95 to 3.05	0.72	0.39 to 1.35	Referent	
ER^-^	276	1.09	0.84 to 1.35	0.50	1.48	0.38 to 5.76	0.81	0.21 to 3.09	Referent	

25(OH)D, 25-hydroxyvitamin D; BMI, body mass index; CI, confidence interval; ER, estrogen receptor; HR, hazard ratio; HRT, hormone replacement therapy.

^a ^Stratified by age at diagnosis and season, adjusted for tumor size, nodal status, metastases, tumor grade, estrogen/progesterone receptor status, diabetes, mode of detection.

^b ^Calculated by using serum 25(OH)D as a continuous variable.

^c ^Calculated by entering in the model the cross-product of 25(OH)D as a continuous variable with the covariate as a binary variable. Cutpoints for BMI and leisure time physical activity were based on median values.

^d ^Participants with blood collection after start of chemotherapy and with BMI obtained more than one month before/after blood collection were excluded from this analysis stratified by BMI.

## Discussion

To our knowledge, this is the largest cohort study to date investigating the association between vitamin D and breast cancer survival using 25(OH)D as a reliable biomarker and direct indicator of bodily vitamin D stores. We found that postmenopausal breast cancer patients with lower post-diagnostic serum 25(OH)D levels were at a statistically significant increased risk of distant recurrence. An association for risk of death was statistically significant only for participants that did not receive chemotherapy before blood collection.

Some circumstantial evidence for a role of vitamin D in cancer survival exists [6], but the association between measured serum 25(OH)D concentrations and breast cancer recurrence and survival has only been investigated in two previous studies [12,13]. These studies were conducted in Canada [12] and the USA [13] and levels of 25(OH)D were relatively high compared with those in our study, probably due to milk fortification with vitamin D and higher use of supplements containing vitamin D. One study in 512 breast cancer patients collected blood on average 58 days after diagnosis and before initiation of systemic therapy [12]. In this study, serum 25(OH)D concentrations less than 50 nmol/L compared with more than 72 nmol/L were associated with an increased risk of distant recurrence and death. Associations were attenuated after multivariate adjustment for tumor-related factors. In a matched case-control analysis in 512 pairs of breast cancer patients with and without a recurrence, blood was collected approximately two years after diagnosis and after completion of treatment for breast cancer [13]. When comparing serum 25(OH)D concentrations less than 25 nmol/L to 75 nmol/L or above, no association with breast cancer recurrence was observed.

The relevant time period during which 25(OH)D levels may affect breast cancer recurrence or survival is currently unknown. The results of our study together with findings of the two previous studies could indicate that serum 25(OH)D concentrations measured shortly after diagnosis are associated with breast cancer recurrence and survival. However, the null association for serum 25(OH)D measured at least two years after diagnosis [13] could not be assessed in our study due to small numbers, and deserves further investigation.

Our results of a significantly poorer survival associated with lower 25(OH)D levels were restricted to participants not receiving chemotherapy before blood collection, which is comparable with those of the Canadian study [12]. Adjuvant chemotherapy may affect 25(OH)D levels, either directly or indirectly by inducing nausea and subsequently reducing dietary vitamin D intake or by decreasing physical activity or sun exposure. One [18] out of three studies [18-20] showed an effect of chemotherapeutic treatment on 25(OH)D levels in breast cancer patients, but study sizes were small (*n *= 20, *n *= 9, and *n *= 103, respectively) and in one study [20] vitamin D supplementation was used. In our study, a larger proportion of women in the lowest compared with the highest tertile of 25(OH)D levels received chemotherapy, and 25(OH)D levels were somewhat lower in participants with blood collected after start of chemotherapy compared with those with blood collected before start of chemotherapy or not receiving chemotherapy (data not shown). Examination of the effect of chemotherapy on 25(OH)D levels in more detail would be of great interest.

Serum 25(OH)D levels have been associated with tumor-related and lifestyle-related factors, and the observed association in our study may not be due to an independent effect of 25(OH)D but instead be reflective of another factor related to 25(OH)D. The current study and several other studies [10-13] showed that lower 25(OH)D levels are related to more advanced tumor stage at diagnosis. Further, vitamin D metabolism is disrupted in breast cancer progression [21]. Our analyses were adjusted for all relevant tumor characteristics, but we cannot exclude potential residual confounding. Lower serum levels of 25(OH)D have also been associated with higher BMI and lower physical activity [22], which was confirmed by our study. However, after restriction to patients with BMI reported within one month of blood collection and exclusion of patients with blood collection after start of chemotherapy, adjustment for BMI did not substantially change the risk estimates. We had no data on physical activity at time of blood collection, however, adjustment for leisure time physical activity since age 50 years did not affect the risk estimates. Thus, the observed association of serum 25(OH)D with survival after breast cancer appeared to be independent of BMI and physical activity although we cannot exclude confounding by potential changes in these factors during follow up. However, stratification of our analyses by tumor stage provided similar results (data not shown), indicating that 25(OH)D levels are associated with prognosis independent of disease severity or overall well being.

We did not observe effect modification by other potential predictors of breast cancer recurrence and mortality, such as BMI, physical activity, HRT use, and ER status. However, our study may not have had sufficient statistical power to detect moderate effects. Potential effect modification by BMI is of particular interest, and has been observed in a previous study on prediagnostic 25(OH)D levels in colorectal cancer survivors [23]. A possible explanation may be that besides its anticarcinogenic properties, 25(OH)D has anti-inflammatory properties that may have an additional beneficial effect on the inflammatory state that is characteristic for obese individuals [23]. However, further investigation in larger studies is warranted.

Strengths of our study are the restriction to postmenopausal women, the population-based design, complete follow up, and detailed data on many potential confounders (tumor characteristics, therapy, lifestyle factors). However, similar to other observational studies, we only had a single measurement of 25(OH)D, which may not be reflective of long-term 25(OH)D concentrations, limited power to detect interactions with other vitamin D-related lifestyle factors, and we cannot exclude residual or uncontrolled confounding.

## Conclusions

The results of our study suggest that lower serum 25(OH)D concentrations after a diagnosis with postmenopausal breast cancer are associated with poorer overall survival and distant disease-free survival. The association with overall survival might be restricted to patients not receiving chemotherapy before blood collection. The relevant time frame of and the effect of chemotherapy on serum 25(OH)D measurements in breast cancer survival studies deserves further investigation.

## Abbreviations

25(OH)D: 25-hydroxyvitamin D; 1,25(OH)_2_D: 1,25-dihydroxyvitamin D; BMI: body mass index; CI: confidence interval; ER: estrogen receptor; HER2: human epidermal growth factor receptor 2; HR: hazard ratio; HRT: hormone replacement therapy; MARIE: Mamma Carcinoma Risk factor Investigation; MET: metabolic equivalent; PR: progesterone receptor.

## Competing interests

The authors declare that they have no competing interests.

## Authors' contributions

AV participated in the follow up field work, carried out the data analysis and drafted the manuscript. RH contributed to the data analysis. SA carried out the 25(OH)D measurements. AS supported the follow up field work and contributed to clinical aspects and data interpretation. DFJ participated in the design of the study and coordination in the Hamburg study region. JCC conceived the study, participated in its design and coordination, and helped to draft the manuscript. All authors read and approved the final manuscript.
